# Primary Malignant Melanoma of the Rectum: Report of Two Cases

**DOI:** 10.1155/2012/247348

**Published:** 2012-12-18

**Authors:** Kodai Tomioka, Hitoshi Ojima, Makoto Sohda, Akiko Tanabe, Yasuyuki Fukai, Akihiko Sano, Takahiro Fukuda, Masahiko Murakami

**Affiliations:** ^1^Department of Gastroenterological and General Surgery, Showa University Hospital, 1-5-8 Hatanodai, Shinagawa, Tokyo 142-8666, Japan; ^2^Department of Gastroenterological Surgery, Gunma Prefectural Cancer Center, 617-1 Takabayashi-Nishi, Ota, Gunma 373-8550, Japan

## Abstract

We report two cases of rectal malignant melanomas. The patients were an 84-year-old male and a 66-year-old female who had blood in their stools. They were preoperatively diagnosed with poorly differentiated adenocarcinoma of the rectum. The clinical diagnosis for each was rectal carcinoma at stage IIIc according to the tumor-node-metastasis classification (6th edition), and the patients underwent abdominoperineal resection with dissection of lymph nodes. Pathological examination of the resected specimens revealed a malignant melanoma. Immunohistochemical analysis results were positive for HMB-45 and negative for cytokeratin AE1/AE3, CD45, and synaptophysin. Primary anorectal melanoma is an uncommon and aggressive disease that carries a poor prognosis. Therefore, it is necessary to provide systemic treatment. To improve prognosis, it is important to detect anorectal melanoma at an early stage.

## 1. Introduction

Anorectal melanoma is an uncommon and aggressive disease. The anorectum is the third most common location of malignant melanoma after the skin and retina. The most common symptom is rectal bleeding, which is often mistaken for bleeding associated with hemorrhoids. Diagnosis is very difficult, and initial diagnosis may be incorrect in 80% of all cases [1, 2]. For patients with anorectal malignant melanoma, treatment strategy includes surgery, chemotherapy, and radiotherapy. However, the tumor tends to be considerably resistant to radiotherapy and shows a poor response to chemotherapy. The choice of wide local excision (WLE) or abdominoperineal resection (APR) is also controversial [3–6]. The prognosis is very poor, with less than 20% survival five years after diagnosis [4, 5, 7]. We present two cases of rectal malignant melanoma with a rapid and fatal course that could not be diagnosed preoperatively.

## 2. Case Presentation

### 2.1. Case  1

**Figure 1:**
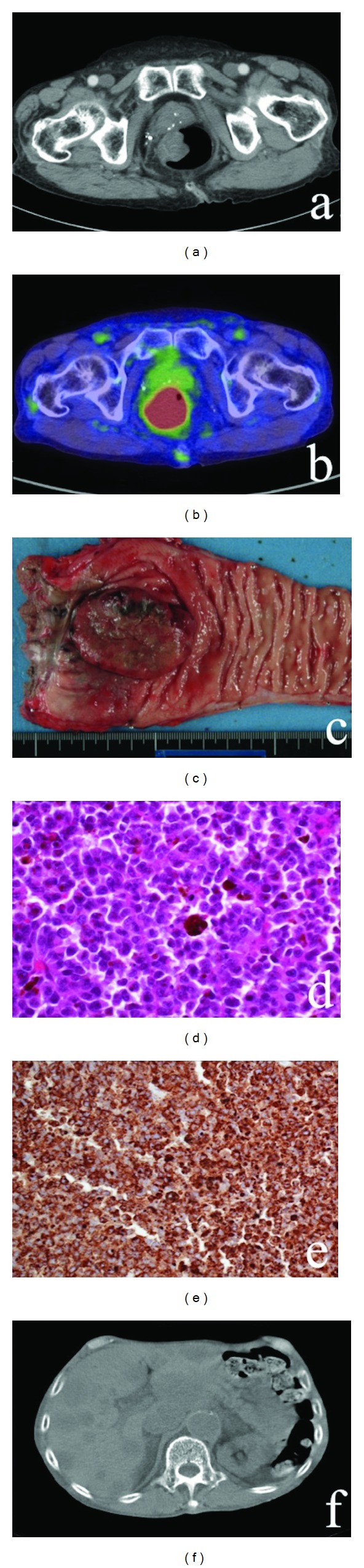
Case  1 CT, FDG-PET, MRI, and pathological imaging. (a) CT shows that the tumor into the lumen. (b) FDG-PET shows that increased accumulation of FDG. (c) Macroscopic image of the rectal tumor showing pigmented lesions. (d) Histopathological examination of the rectal specimen showed the nest of melanocytic cells (e.g., HE stain, ×40). (e) Rectal specimen is positive for the expression of HMB-45 (×20). (f) CT shows multiple liver metastases.

An 84-year-old male was referred to our hospital with the chief complaint of bloody stool. Digital examination of the rectum revealed a hard mass at the 9 o'clock position. Colonoscopy revealed an irregular surface mass with a diameter of approximately 60 mm, located on the right wall of the lower rectum, 30 mm from the anal verge. Biopsy of the rectal mass was performed, and histopathological examination showed poorly differentiated adenocarcinoma. Computed tomography (CT) showed a thickening of the rectal wall and lymph node swelling of the circumference of an internal iliac artery; however, there was no evidence of distant metastasis (Figure 1(a)). Magnetic resonance imaging (MRI) also showed thickening of the rectal wall and enlarged regional lymph nodes. Diffusion-weighted imaging (DWI) produced a high signal, and ^18^F-fluorodeoxyglucose positron emission tomographic (FDG-PET) imaging revealed a soft mass with increased accumulation of FDG (Figure 1(b)). The standardized uptake value of the main tumor was 19.25. Laboratory data as well as serum carcinoembryonic antigen (CEA) and CA19-9 levels were almost normal.

The clinical diagnosis was rectal carcinoma at stage IIIc according to the tumor-node-metastasis (TNM) classification (6th edition). The patient was treated by abdominoperineal resection (APR) with dissection of lymph nodes. The resected specimen showed some pigmented lesions within the tumor and around the anal verge (Figure 1(c)). Histopathological examination of the specimen showed a pattern of pleomorphic cells with melanin pigmentation of the cytoplasm (Figure 1(d)). Immunohistochemical analysis results were positive for the expression of S-100 protein and HMB-45 (Figure 1(e)) and negative for the expression of cytokeratin AE1/AE3, CD45, and synaptophysin. The final diagnosis was malignant melanoma. The patient was discharged on the 21st postoperative day after an uneventful course. Postoperative adjuvant chemotherapy was not performed because of advanced age.

Three months after the resection, the patient was rehospitalized with a chief complaint of right leg edema and dyspnea. Laboratory data showed liver and renal dysfunction. CT showed multiple liver and right inguinal lymph node metastases (Figure 1(f)). The patient died of hepatic insufficiency three days later.

### 2.2. Case  2

**Figure 2:**
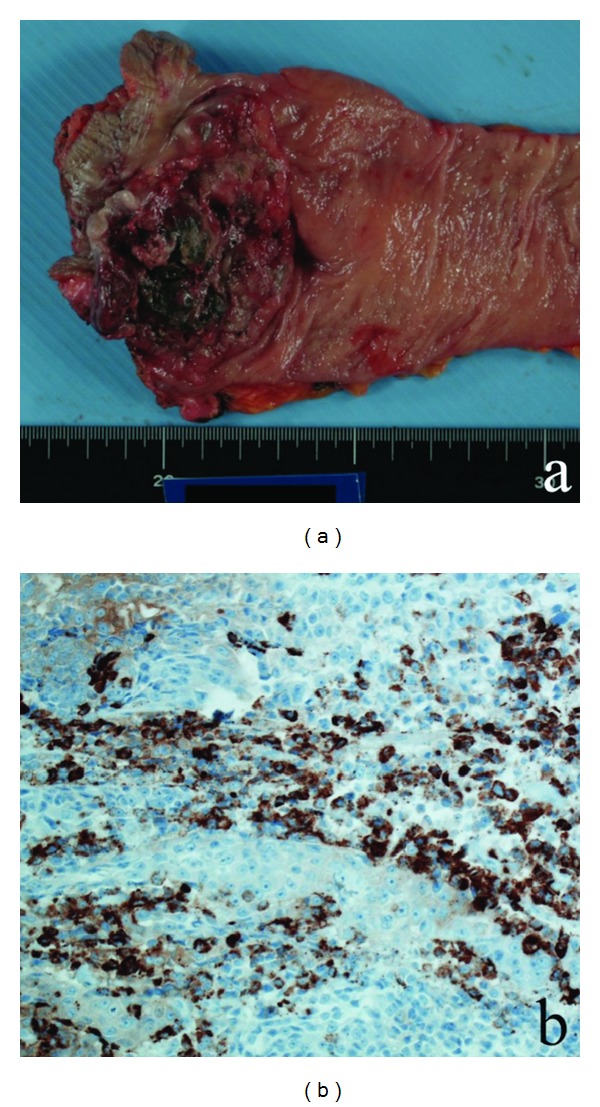
Case  2 pathological imaging. (a) Macroscopic image of the rectal tumor showing some pigmented lesions. (b) Rectal specimen is positive for the expression of HMB-45 (×20).

 A 66-year-old female was referred to our hospital with the chief complaint of blood in the stool. Digital examination revealed a hard mass located all around the wall, 1.0 cm from the anal verge. Colonoscopy revealed an irregular surface large mass of approximately 80 mm in diameter located all around the wall of the lower rectum, 10 mm from the anal verge. Histopathological examination showed features of poorly differentiated adenocarcinoma. CT and MRI revealed an increased density in the perirectal area, rectal wall thickening all-around, and regional lymph node metastases. DWI produced a high signal in the previously described area. Laboratory data as well as CEA and CA19-9 levels were almost normal. The boundary with the vaginal wall was unclear. Under the diagnosis of stage IIIc rectal carcinoma according to the TNM classification (6th edition), we performed an APR with dissection of lymph nodes. Because there was invasion of the vagina, a part of the vagina was also resected at the same time. The resected specimen revealed some pigmented lesions within the tumor and around the anal verge (Figure 2(a)). The patient was discharged on the 31st postoperative day after an uneventful course.

Histopathological examination showed the characteristics of malignant melanoma. Immunohistologically, the results showed positive expression of HMB-45 (Figure 2(b)) and cytokeratin AE1/AE3 and negative expression of S-100 protein, CD45, and synaptophysin. Postoperative adjuvant chemotherapy of dacarbazine (DTIC) was given. Bleeding from the vagina occurred one month after leaving the hospital, and a local recurrence was detected. Two months later, liver, lung, and brain metastases were detected. The chemotherapy appeared to be ineffective in preventing disease progression. The patient died of hepatic insufficiency and disseminated intravascular coagulation six months after the resection.

## 3. Discussion

Malignant melanoma of the rectum is rare and has very poor prognosis. The incidence has been reported to be 0.4%–3.0% of all malignant melanoma and 0.1%–4.6% of all anorectal malignant tumors [4, 8–10]. Melanomas of the anorectum are the third most common after melanomas of the skin and retina. Malignant melanomas occur frequently in the anorectum because of the presence of abundant melanocytes in the mucosa of the anal canal. The reported 5-year overall survival rate is 6%–15% of patients after surgery [4, 5, 7, 11–13]. Several studies have reported cases of long-term survival [14–16]. The main determinants of prognosis are the depth of invasion and stage of the disease [17]. Early-stage detection is important. The tumor has been reported in older patients and women, and the common initial symptoms are rectal bleeding and/or pain. Obvious melanin pigmentation is present in only 20% of patients [18]. Therefore, the symptoms are often confused as those of hemorrhoids. Nonspecific symptoms cause delayed diagnosis, which is also caused by the similarity of histological findings to those of other malignancies. The clinical diagnosis may be incorrect in 80% of all cases [1, 2, 18]. Because of delayed diagnosis and rapid progression, malignant rectal melanomas have been accompanied by distant metastases in 60% of patients at the time of final diagnosis [4, 13]. The two present cases had a chief complaint of rectal bleeding, and clinical diagnosis before surgery was rectal carcinoma. Although curative surgery was performed for these cases, their disease had already advanced to stage IIIb. Preoperative biopsy of the tumors showed poorly differentiated adenocarcinoma, which was different from the final diagnosis. Immunohistochemical studies are useful methods for establishing correct diagnosis, and the diagnoses in our cases were confirmed by the expressions of S-100 protein and HMB-45. In these cases, the final diagnosis was based on immunohistochemical studies.

For anorectal malignant melanoma, multimodality treatments including surgery, chemotherapy, and radiotherapy have been used. Surgery is the main treatment. The surgical procedure varies from WLE to APR. However, the relative benefit of these individual procedures is unclear [5, 19]. In our cases, APR was performed because the preoperative diagnosis was poorly differentiated adenocarcinoma, and it was possible to perform curative surgery. There are some reports that these surgical therapies have minimal impact on prognosis, but they can have some effect in controlling symptoms or improving the patient's quality of life. Correlation between the depth of invasion and median survival has also been reported [17], and long-term survival is possible after curative surgery [14–16]. Therefore, we should choose surgical procedures according to the tumor stage. 

The tumor tends to be quite radiotherapy resistant and shows a poor response to chemotherapy [16]. The role of adjuvant chemotherapy has not been established. The prognosis is poor regardless of any therapies, and the most important predictors of prognosis are disease stage, symptom duration, tumor size, and nodal status [15, 20, 21]. Therefore, early detection of anorectal melanoma is critical for reducing the mortality rate.

In conclusion, anorectal melanoma is a rare and aggressive disease. Because of nonspecific symptoms, it is easily mistaken for hemorrhoids. Because malignant melanoma occurs frequently in the anorectum, clinicians should suspect anorectal melanoma in cases presenting with blood in the stool. Furthermore, the prognosis depends on the staging, and it is important to detect anorectal melanoma at an early stage. Ultimately, the development of further effective adjuvant therapy may improve the survival rate.
